# Guiding resuscitation in shock: base excess or lactate?

**DOI:** 10.1186/s13054-024-05039-2

**Published:** 2024-07-18

**Authors:** Micah Liam Arthur Heldeweg, Thomas Langer, František Duška

**Affiliations:** 1 Department of Intensive Care Medicine, Amsterdam University Medical Centers, Amsterdam, The Netherlands; 2 Department of Anaesthesia and Intensive Care Medicine, The Third Faculty of Medicine, Charles University, FNKV University Hospital, Prague, Czech Republic; 3 Department of Medicine and Surgery, University of Milan-Bicocca, Monza, Italy; 4 Department of Anesthesia and Intensive Care Medicine, Niguarda Ca’ Granda, Milan, Italy

**Keywords:** Base excess, Base deficit, Lactate, Metabolic acidosis, Resuscitation, Fluid management

Base excess (BE) is a widely used parameter derived from blood gas analysis. A recent international study showed that 40% of surveyed anesthesia and critical care clinicians use BE to guide (intraoperative) fluid management, and that 25% of respondents prefer BE over lactate [1]. This is surprising as lactate production is directly increased by hypovolemia-associated tissue hypoxia, whilst BE is a simple calculation of the metabolic component of acid-base derangement.

In the 1960s, measuring lactate was laborious and time-consuming: a typical colorimetric measurement of lactate took up to eight hours, clearly limiting its clinical point-of-care use [2]. At the same time, Astrup and Siggaard-Andersen introduced BE, a marker to quantify metabolic acid-base derangements independent of concomitant carbon dioxide variations, i.e. respiratory acid-base disorders. Modern BE is a simple mathematical expression that corrects changes in bicarbonate for carbon dioxide variation by using the slope of an experimentally determined carbon dioxide titration curve [3].

In contrast to lactate, BE was able to provide an instant and inexpensive quantification of a metabolic acidosis, occurring, for example, during circulatory shock. Unsurprisingly, in this context, many researchers considered BE a surrogate indicator of oxygen debt and hypovolemia. BE was ideal in the predominantly healthy traumatology population: a single surrogate marker for hemorrhagic shock severity that could be used as a therapeutic trigger for transfusion management. Investigations demonstrated that BE was more accurate at quantifying the magnitude of blood loss during hemorrhagic shock than clinicians’ visual estimates of blood loss, volume replacement counts, blood pressure, or heart rate [4].

Development of refined electrode-based lactate measurement methods enabled direct and rapid assessment, starting the era of routine lactate measurement in the 1980s [2]. Nonetheless, some authors deemed lactate as clinically less useful than BE because of potential confounding factors: inflammation, sympathetic stimulation, drugs such as metformin, hepatic failure, and exogenous lactate may all lead to a hyperlactatemia in the absence of oxygen debt [4]. Moreover, lactate normalization depends on its clearance and may be delayed despite effective resuscitative measures. Indeed, hyperlactatemia may reflect other pathophysiological mechanisms unrelated to hemorrhagic shock [5]. However, any increase in endogenous lactate, by definition, leads to a decrease in BE. Moreover, BE is a composite marker and may be influenced by more factors than only hyperlactatemia: changes in strong electrolytes, such as sodium and chloride, weak acids, such as albumin or phosphate, and other unmeasured acids, such as ketones and toxins. Notably, resuscitation with a 0.9% saline solution leads to a hyperchloraemic metabolic acidosis with a decreased BE [3].

Nonetheless, research in traumatology continued to employ BE. A landmark *Critical Care* cohort study heralded ‘the renaissance of BE’: investigators found that BE may be superior to traditional vital parameters (heart rate, systolic blood pressure, and Glasgow coma scale) for identification of transfusion requirements in traumatology patients [3]. BE was subsequently included in the guidelines and the ATLS classification of hypovolemic shock, an internationally-used teaching and management instrument for resuscitation. This established a paradigm in traumatology and resuscitation management that permeated into emergency medicine, anesthesia, and critical care [1]. Unfortunately, the original study did not perform a direct comparison of the performance of lactate versus BE to predict transfusion requirements [3].

Meanwhile, routine point-of-care testing spread across emergency and critical care settings worldwide, and mounting evidence supported the value of serial lactate measurements in the evaluation of critically ill patients and their response to (fluid) therapy. Clinicians widely embraced lactate as the best-available single high-sensitivity indicator of shock severity and a central therapeutic trigger in resuscitation protocols. In critical care, appraising lactate jointly with central mixed venous oxygen saturation may (partially) offset its limited specificity [5].

Currently lactate, an underlying metabolic substrate of tissue hypoxia, can be directly, reliably, and serially measured [2]. Considering BE its surrogate is, in our opinion, anachronistic. However, as these parameters provide different information, their joint evaluation remains informative. Indeed, if BE cannot be fully explained by hyperlactatemia, there may be a concurrent acid-base derangement, which should be identified and addressed. The presence of an ‘alactic’ BE may therefore be etiologically and prognostically valuable in traumatology, and perhaps, critically ill patients [1, 4]. There is a burning need for studies directly comparing the utility of metabolic parameters such as BE, lactate and alactic BE as tailored resuscitation trigger and prognostic markers in acute hypovolemia.

In conclusion, the current literature does not advocate for a single, optimal metabolic resuscitation trigger. Clinicians should integrate multiple metabolic variables with clinical parameters to decide the resuscitation strategy and evaluate its effect. Until direct comparisons are available, there is good rationale to suggest that, mechanistically, lactate is a more appropriate resuscitation trigger, whilst BE remains useful in identifying acid-base derangements superimposed on lactic acidosis.

## Data Availability

No datasets were generated or analysed during the current study.
